# The SNF2 family ATPase LSH promotes cell-autonomous *de novo* DNA methylation in somatic cells

**DOI:** 10.1093/nar/gkw424

**Published:** 2016-05-13

**Authors:** Ausma Termanis, Natalia Torrea, Jayne Culley, Alastair Kerr, Bernard Ramsahoye, Irina Stancheva

**Affiliations:** 1 Wellcome Trust Centre for Cell Biology, University of Edinburgh, Michael Swann Building, Max Born Crescent, Edinburgh EH9 3BF, UK; 2 Institute of Genetics and Molecular Medicine, University of Edinburgh, Western General Hospital, Crewe Road South, Edinburgh EH4 2XR, UK

## Abstract

Methylation of DNA at carbon 5 of cytosine is essential for mammalian development and implicated in transcriptional repression of genes and transposons. New patterns of DNA methylation characteristic of lineage-committed cells are established at the exit from pluripotency by *de novo* DNA methyltransferases enzymes, DNMT3A and DNMT3B, which are regulated by developmental signaling and require access to chromatin-organized DNA. Whether or not the capacity for *de novo* DNA methylation of developmentally regulated loci is preserved in differentiated somatic cells and can occur in the absence of exogenous signals is currently unknown. Here, we demonstrate that fibroblasts derived from chromatin remodeling ATPase LSH (HELLS)-null mouse embryos, which lack DNA methylation from centromeric repeats, transposons and a number of gene promoters, are capable of reestablishing DNA methylation and silencing of misregulated genes upon re-expression of LSH. We also show that the ability of LSH to bind ATP and the cellular concentration of DNMT3B are critical for cell-autonomous *de novo* DNA methylation in somatic cells. These data suggest the existence of cellular memory that persists in differentiated cells through many cell generations and changes in transcriptional state.

## INTRODUCTION

Methylation of DNA at the fifth carbon of cytosine (5mC) is an abundant epigenetic modification in vertebrate genomes (1). In mammals, DNA methylation is established during development and contributes to regulation of genomic imprinting, tissue-specific gene expression, silencing of retrotransposons and X chromosome inactivation in females (2,3). The deposition of new methyl groups to cytosine occurs by the action of two homologous enzymes, the *de novo* DNA methyltransferases DNMT3A and DNMT3B, while the propagation of 5mC through DNA replication requires the activity of maintenance DNA methyltransferase DNMT1 (4). DNMTs are critical in early mammalian development when, following a nearly global erasure of 5mC during the cleavage stages of pre-implantation embryo, new patterns of 5mC are established post-implantation in the developing epiblast (E6.5) (3,5,6). Embryos lacking either DNMT1 or DNMT3B display severe 5mC deficiency and die at mid-gestation (E9.5–E11) (7,8). Several studies have identified DNMT3B as the main enzyme responsible for *de novo* DNA methylation during development (6,8–10). In *Dnmt3b−/−* embryos, the centromeric repeats, promoters of germ cell-specific genes and genes on the inactive X chromosome in female embryos remain hypomethylated.

The occurrence of new methylation at specific time of development suggests that the levels and the activity of DNMTs must be tightly controlled and coupled to developmental signaling. Several signal transduction pathways, in particular FGF and WNT, have been implicated in the exit from pluripotency, priming of embryonic cells for differentiation and regulation of DNA methylation. Thus simultaneous inhibition of mitogen-activated protein kinase (MAPK) and glycogen synthase kinase 3 (GSK3) pathways by specific inhibitors (2i) reinforces the naïve pluripotency of embryonic stem (ES) cells and this is accompanied by rapid downregulation of DNMT3B and loss of 5mC (11–13).

In addition to developmental signaling, the activity of DNMTs is also regulated at the level of chromatin. Unlike DNMT1 that methylates newly replicated hemimethylated DNA largely devoid of nucleosomes, the DNMT3 enzymes must function on DNA organized into chromatin. In comparison to naked DNA, stably positioned nucleosomes are a poor substrate for *de novo* DNA methylation *in vitro* and partly *in vivo* (14,15). Therefore the efficient *de novo* methylation of chromatin-organized DNA in cells and embryos requires either dynamic repositioning of nucleosomes or loosening of the contacts between the histones and DNA. In agreement with this, several ATP-dependent chromatin remodeling enzymes have been implicated in the regulation of 5mC levels and patterns, including the mammalian SNF2 family ATPases ATRX and LSH (16,17). A knockout of *Lsh* (*Hells*) gene in mice results in ∼50% reduction of the global 5mC in the genome affecting repetitive sequences and large chromosomal domains associated with the nuclear lamina (16,18,19). Mapping of 5mC at promoters of protein-coding genes in wild-type and *Lsh−/−* mouse embryonic fibroblasts (MEFs) detected loss of 5mC from 20% of normally methylated promoters (19), many of which undergo lineage-specific silencing and *de novo* DNA methylation during early mouse development (10). Importantly, many of these genes are inappropriately expressed in the *Lsh−/−* MEFs (19). As DNMTs are present at normal levels in LSH-deficient cells (16) and LSH interacts directly with DNMT3B (20), these findings suggest that ATP-dependent chromatin remodeling is critical during development to open up chromatin for developmentally programmed DNA methylation by *de novo* enzymes.

If the programmed *de novo* DNA methylation were tightly regulated by signaling pathways in the developing embryo, one would predict that the loss of 5mC would be irreversible in somatic cells taken out of their normal developmental context. In order to investigate whether this is the case, we restored the expression of LSH in spontaneously immortalized hypomethylated *Lsh−/−* MEFs grown in culture for many cell generations. Contrary to our expectations, we found that 5mC at repetitive and unique sequences as well as gene silencing of developmentally regulated loci could be substantially reestablished when a wild-type LSH protein was introduced into the *Lsh−/−* MEFs. We also found that the reversal of 5mC levels and patterns in the *Lsh−/−* MEFs required the catalytic activity of LSH ATPase and appropriate cellular concentration of DNMT3B. Taken together, these experiments demonstrate that the capacity for LSH-regulated *de novo* DNA methylation of repetitive sequences and transcriptionally active developmentally regulated promoters is preserved in somatic cells. These experiments also suggest the existence of epigenetic cellular memory, which persists through changes in transcriptional state and permits silencing of inappropriately expressed genes and repetitive sequences in the absence of developmental signaling.

## MATERIALS AND METHODS

### Cell culture

All mouse embryonic fibroblast cell lines were grown in DMEM (Sigma) supplemented with 10% fetal bovine serum (Thermo Fisher), 100 units/ml penicillin, 1 mg/ml of streptomycin and 2 mM l-glutamine.

### Establishment of stable cell lines

pMSCV-LSH and pMSCV-K237Q plasmids were generated by cloning into pMSCV vectors carrying either puromycin or hygromycin resistance marker of mouse synthetic LSH ORF (wild-type and mutant; Life Technologies/GeneART) designed to contain an in-frame C-terminal 3xFLAG tag followed by the 3′UTR and polyadenylation sequence of the LSH mRNA. pMSCV, pMSCV-LSH and pMSCV-K237Q plasmids were packaged into lentiviral particles in Phoenix A cells. Culture supernatants were harvested 48 h post-transfection and the lentiviral titres determined by infection of NIH-3T3 mouse fibroblasts. 3 × 10^5^ *Lsh−/−* MEFs were infected with lentiviral particles at MOI = 1 in the presence of 4 μg/ml Polybrene. The cells were transferred to larger dishes 48 h post-infection and selected with 2.5 μg/ml puromycin for 7–10 days. Individual colonies were isolated and expanded into cell lines. DNMT3B was knocked down by infecting the *Lsh−/−* MEFs with packaged lentiviral particles (Thermo Scientific) carrying pGIPZ plasmids with either non-silencing shRNAmir (V3LMM_420607) or shRNAmir sequences targeting murine DNMT3B mRNA (V2LMM_257108; V2LMM_257108; V2LMM_53727) at MOI = 3 in the presence of 4 μg/ml Polybrene. The cells were selected with 2.5 μg/ml puromycin for 7–10 days and subsequently infected with lentiviral particles carrying pMSCV-LSH-hygro at MOI = 1. The cells were selected with 400 μg/ml hygromycin and single colonies expanded into cell lines.

### Western blots

Nuclear extracts were prepared as described (19) and 60 μg of each extract were resolved in 7% SDS-polyacrylamide gel. The gels were transferred to nitrocellulose membrane (BioRad), blocked with 4% skimmed milk in 1 × TBS; 0.1% Tween and incubated with primary antibodies in blocking solution overnight. The membranes were washed extensively with PBS; 0.1% Tween, re-blocked for 30 min and incubated with secondary anti-mouse IR-800 and anti-rabbit IR-670LT antibodies (LiCOR Biosciences) for 1 h. After washing with PBS; 0.1% Tween, the membranes were imaged on Odyssey scanner (LiCOR Biosciences) and quantified when required with Image Studio Software (LiCOR Biosciences). The primary antibodies used for Western blots were: anti-LSH (Santa Cruz; sc-46665), anti-Flag M2 (Sigma), anti-MRE11 (Calbiochem; PC388), anti-HDAC1 (Santa Cruz; sc-7872) and anti-DNMT3B (Thermo Fisher; AFPA1884).

### Southern blots

Genomic DNA was purified from MEF cell lines using standard protocols and 10 μg of each DNA were digested with either MspI or its methylation-sensitive isoschizomer HpaII for 3 h at 37°C. DNA was resolved on 1% Agarose gels run in 1 × TAE buffer, the gels were transferred to nitrocellulose membrane (Pall B; VWR) with 0.4 M NaOH and hybridized with minor-satellite probe labeled with α-P^32^-dCTP (Perkin Elmer) by a random-priming kit (Thermo Scientific) in buffer containing 1 mM EDTA, 0.5 M NaHPO4 and 7% SDS at 65°C overnight. The blots were subsequently washed with 3× SSC; 0.1% SDS at 65°C and imaged on Typhoon phosphorimager (GE Healthcare).

### Bisulfite DNA sequencing

For bisulfite DNA sequencing, genomic DNA was processed with EpiTect Bisulfite conversion kit (QIAGEN) and used as a template for PCRs with specific primer pairs, which are listed in Supplementary Table S1. PCR products were cloned into pJet vector (Thermo Scientific) and sequenced using a reverse pJet primer and BigDye sequencing mix (Applied Biosystems). Sequences were analyzed by BiQ Analyzer software (21).

### Analyses of DNA methylation by reverse-phase HPLC

DNA was extracted from ESCs following standard protocols and residual RNA was removed by enzymatic hydrolysis (6-hour incubation with RNase A and RNase T1) followed by DNA precipitation in 3 volumes of ethanol. This was repeated once. 1–5 μg of purified DNA was digested with DNase1 (New England Biolabs) for 12 h in the recommended buffer. Following this, 2 volumes of 30 mM sodium acetate pH 5.2 was added and the DNA was further digested to nucleotide 5′ monophosphates with Nuclease P1 (Sigma) in the presence of 1 mM zinc sulphate (7-h incubation). The quantitation of 5-methylcytosine in genomic DNA was performed in triplicate by isocratic high performance reverse phase liquid chromatography as previously described (22), with the following alterations. A Dionex UM 3000 HPLC system was used complete with a column chiller, C18 column (250 mm × 4.6 mm 5 um APEX ODS, #4M25310, Grace Discovery Sciences), and column guard (Phomenex, #AJ0-7596). The mobile phase was 50 mM ammomium phosphate (monobasic) pH4.1. The column was chilled to 8°C to improve peak separation. Deoxyribonucleotides (dNMPs) were detected at their extinction maxima using a Dionex 3000 multiple wavelength detector: dCMP, 276 nm; 5mdCMP, 282 nm. Quantifications were calculated from the area under each peak using the respective extinction coefficients (dCMP, 8.86 × 10^3^; 5mdCMP 9.0 × 10^3^).

### Quantitative RT-PCR

Total RNA was purified from cell lines using TRIzol reagent (Life Technologies) according to manufacturer's instructions and cDNA synthesis performed with Superscript II (Life Technologies). Quantitative reverse transcription PCRs were carried out in three biological replicates with six technical replicates for each with SYBR-Green Mastermix (Roche) on Roche LC480 instrument. Fold changes relative to *GAPDH* were calculated using the Pfaffl method (23). Primer sequences are listed in Supplementary Table S1.

### Chromatin immunoprecipitation

Chromatin immunoprecipitation was performed essentially as described in (19). Briefly, the cells were crosslinked with 1% formaldehyde in DMEM supplemented with 10× crosslinking buffer (500 mM HEPES, pH7.9; 1.5 M NaCl; 10 mM EDTA; 5 mM EGTA), to 1× final concentration. After neutralising the formaldehyde with 2.5 M glycine (final concentration 125 mM), the cells were spun at 1200 rpm at 4°C and washed with PBS supplemented with PMSF. The crosslinked cells were resuspended and incubated in buffer L1 (50 mM HEPES, pH 7.9; 140 mM NaCl; 1 mM EDTA; 10% glycerol; 0.5% NP-40; 0.25% Triton X-100) for 5 min, spun and resuspended in buffer L2 (10 mM Tris pH 8; 200 mM NaCl; 1 mM EDTA; 0.5 mM EGTA). After incubation for 5 min, the cells were spun again, resuspended in TE with 0.3% SDS at density 4 × 10^6^ cells/ml and sheared with BioRuptor sonicator (Diagenode) to fragments of average size 250–300 bp. 10 μg of chromatin were used per IP with 4 μg of either rabbit anti-H3K4me3 antibody (Active Motif) or control rabbit IgG. IP washes were performed as described elsewhere (19). After descrosslinking, immunoprecipitated DNA was purified with PureLink kit (Thermo Fisher) and 1/50 of input and ChIP DNA were used in each 20 μl quantitative PCR reaction. ChIP experiments were performed in two biological replicates with six technical replicates for each. Primers are listed in Supplementary Table S1.

### MeDIP sequencing and data analyses

MeDIP was performed essentially as described (24) with anti-5mC antibody (Eurogentec) on genomic DNA sonicated to average fragment size 300 bp. Ligation of Illumina adaptor sequences was performed prior to MeDIP with NEB-next DNA library Mastermix Set and Singleplex Illumina adaptor oligos (NEB) according to manufacturer instructions. After MeDIP, the DNA was amplified with 14 cycles of PCR and size selected on agarose gel for fragments 250–350 bp. The resulting libraries were Single-end sequenced on Illumina HiSeq2000 instrument generating 120–140 million reads per sample 99% of which could be mapped to the mouse genome. The MeDIP-seq reads were mapped with Burrows Wheeler Algorithm mem version 0.7.5a to the mm10 genome assembly. Sam files were processed into bam files using samtools version 0.1.19. Count tables for each of the studied regions were created using the coverage program in version 2.17.0 of the bedtools suite. Count tables were first normalized to raw reads per kilobase of feature, and then normalized to regions that were previously shown not to change DNA methylation between wild-type and *Lsh−/−* MEFs (19). A linear regression line of the log2 read counts of these non-changing regions was calculated, and the whole dataset was transformed such so that the line would have an intercept of 0 and a gradient of 1. A script written in R (normalise2file.r) can be downloaded from https://github.com/AlastairKerr/Termanis2015. Data was plotted using the ggplot2 package in R. Methylation recovery (MR) was calculated as MR = [100 x (log_2_*D* – log_2_*S*)]/*D* where *D* = log_2_ read count WT - log_2_ read count MSCV and *S* denotes the log_2_ read count for each of the cell lines expressing either LSH or K237Q. The MeDIP-seq data can be accessed at NCBI GEO database using accession number [GEO: GSE64384].

## RESULTS

### *Lsh−/−* MEFs expressing wild-type LSH display an increase in 5mC and methylate repetitive sequences

**Figure 1.**
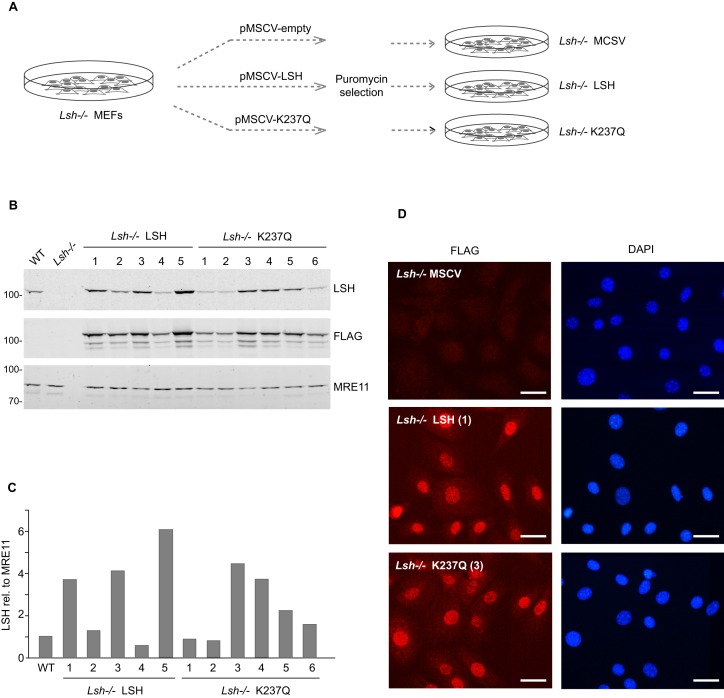
Generation of stable *Lsh−/−* MEF cell lines expressing either wild-type or catalytically inactive LSH. (**A**) Schematic diagram showing the generation of stable cell lines from the *Lsh−/−* MEFs by lentiviral transduction of packaged pMSCV vectors. (**B**) Expression of the wild-type LSH and ATP binding-deficient mutant form, LSH K237Q (K237Q), in clonal cell lines. Quantitative Western blots were probed with the indicated antibodies. MRE11 was used as a loading control. (**C**) Quantification of the wild-type and mutant 3xFLAG-tagged LSH protein levels in clonal cell lines. (**D**) Immunofluorescence staining of stable cell lines with anti-FLAG antibody. The nuclei were counterstained with DAPI. The scale bar corresponds to 20 μm.

To investigate if the reduced levels of 5mC and the misexpression of normally silenced loci (genes and retrotransposons) are reversible in the *Lsh−/−* MEFs and if such reversal requires the catalytic activity of LSH, we introduced by lentiviral transduction a triple FLAG (3xFLAG)-tagged full length LSH, either wild-type or ATP binding-deficient mutant (25), carrying lysine 237 mutated to glutamine (K237Q), into the *Lsh−/−* MEFs. As an additional control, we also infected the LSH-null cells with viral particles carrying an empty vector (pMSCV). As the lentiviral vector carries puromycin resistance marker, we subjected the transduced cells to selection and subsequently isolated stable clonal cell lines (Figure 1A). For convenience, we will refer to these cell lines as *Lsh−/−* LSH, *Lsh−/−* K237Q and *Lsh−/−* MSCV. Quantitative Western blots (Figure 1B and C) and immunostaining with anti-FLAG antibodies (Figure 1D) revealed that most cell lines, except *Lsh−/−* MSCV, stably expressed either the wild-type or mutant LSH, although at different levels, and displayed a relatively homogeneous distribution of the 3xFLAG-LSH throughout the nucleus. We selected four cell lines, two expressing the wild-type LSH (Figure 1B and C, labeled 1 and 3) and two expressing the K237Q mutant (Figure 1B and C, labeled 3 and 4), for further analyses.

**Figure 2.**
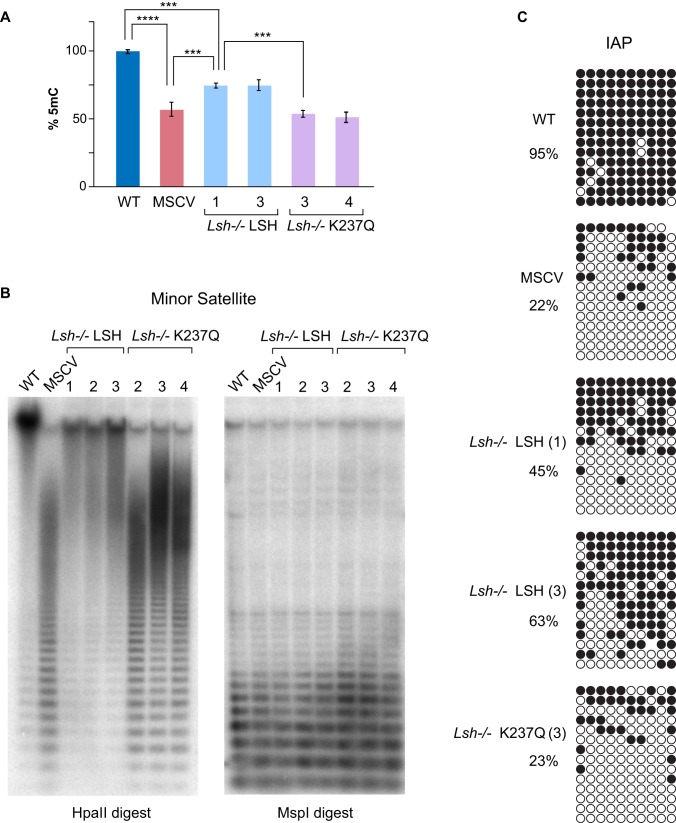
Wild-type LSH restores DNA methylation globally and at repetitive sequences in *Lsh−/−* MEFs. (**A**) Quantification of 5mC by HPLC in indicated cell lines. To simplify the comparison, the amount of 5mC in wild-type MEFs (3.3% of total cytosine) is represented as 100%. Error bars represent standard deviation, *n* = 3, *P* values (*t*-test) **** is *P* < 10^−4^ and *** is *P* < 10^−3^. (**B**) Southern blots of either HpaII (methylation sensitive) or MspI (methylation insensitive) restriction enzymes-digested genomic DNA from indicated cell lines hybridized with ^32^P-labeled centromeric mouse minor satellite probe. (**C**) Bisulfite DNA sequencing of IAP retrotransposons in indicated cell lines. Each row represents a single DNA strand, and each circle is a CpG dinucleotide. Methylated CpGs are shown in black.

To examine if the expression of either LSH or K237Q in the *Lsh−/−* MEFs led to detectable changes in DNA methylation, we analyzed by High Performance Liquid Chromatography (HPLC) the total amount of 5mC in DNA purified from the clonal cell lines and compared the values with those of the controls, *Lsh−/−* MSCV and wild-type MEFs. The HPLC analyses detected a significant increase (∼25%) of 5mC in both *Lsh−/−* LSH cells lines compared to *Lsh−/−* MSCV, but not in the *Lsh−/−* K237Q cell lines (Figure 2A). These data suggest that the wild-type LSH, but not the catalytically inactive K237Q mutant, promotes *de novo* DNA methylation in the *Lsh−/−* MEFs although both proteins and DNMT3B displayed comparable association with chromatin (Supplementary Figure S1).

DNA methylation of most repetitive sequences, including minor (centromeric) and major (pericentric) satellite repeats, IAP retrotransposons and LINE elements, is LSH-dependent (26) (Figure 2B and C, compare WT with MSCV) and some of these sequences are transcribed in the *Lsh−/−*, but not in wild-type MEFs (27,28). To investigate further the patterns of DNA methylation in the *Lsh−/−* MEFs with restored LSH expression, we analyzed DNA methylation at specific repetitive sequences. Southern blots on DNA digested with methylation sensitive HpaII restriction enzyme hybridized with minor satellite probe detected the characteristic ladder of hypomethylated satellite repeats in *Lsh−/−* MSCV MEFs and in three independent *Lsh−/−* K237Q cell lines (Figure 2B). This laddering was largely absent in HpaII-digested DNA derived from *Lsh−/−* LSH cell lines (Figure 2B). Interestingly, there was no significant correlation between the efficiency of methylation at satellite sequences and the expression levels of wild-type LSH in the *Lsh−/−* cell lines (Compare Figure 1B and C with Figure 2B). Furthermore, bisulfite DNA sequencing of IAP retrotransposons detected a significant 20–40% increase in DNA methylation in the *Lsh−/−* LSH cell lines, while methylation of IAPs in *Lsh−/−* K237Q cells was comparable to that of the MSCV control (Figure 2C). Collectively, these analyses demonstrate that the *Lsh−/−* somatic cells are capable of efficient *de novo* DNA methylation of repetitive sequences upon expression of wild-type LSH, but not of the catalytically-inactive K237Q mutant.

### *De novo* gene silencing and DNA methylation in the *Lsh−/−* MEFs require LSH, but can occur in the absence of differentiation signals

As mentioned earlier, about 20% of all promoters normally methylated in wild-type MEFs lack DNA methylation in the *Lsh−/−* MEFs (19). Among these are the promoters of reproductive homeobox (*Rhox*) genes and pluripotency-associated genes (*Dppa2* and *Dppa4*), which undergo transcriptional silencing and programmed DNMT3A/3B-dependent *de novo* DNA methylation during early development in cells that contribute to the embryo proper (10,29). Normally, the *Rhox* genes are expressed and their promoters are not methylated in extraembryonic tissues and in germ cells, but are stably silenced and methylated in the embryo and all somatic cells (10,30). The vast majority of *Rhox* genes are hypomethylated and expressed in *Lsh−/−* MEFs, but not in their wild-type counterparts (19).

**Figure 3.**
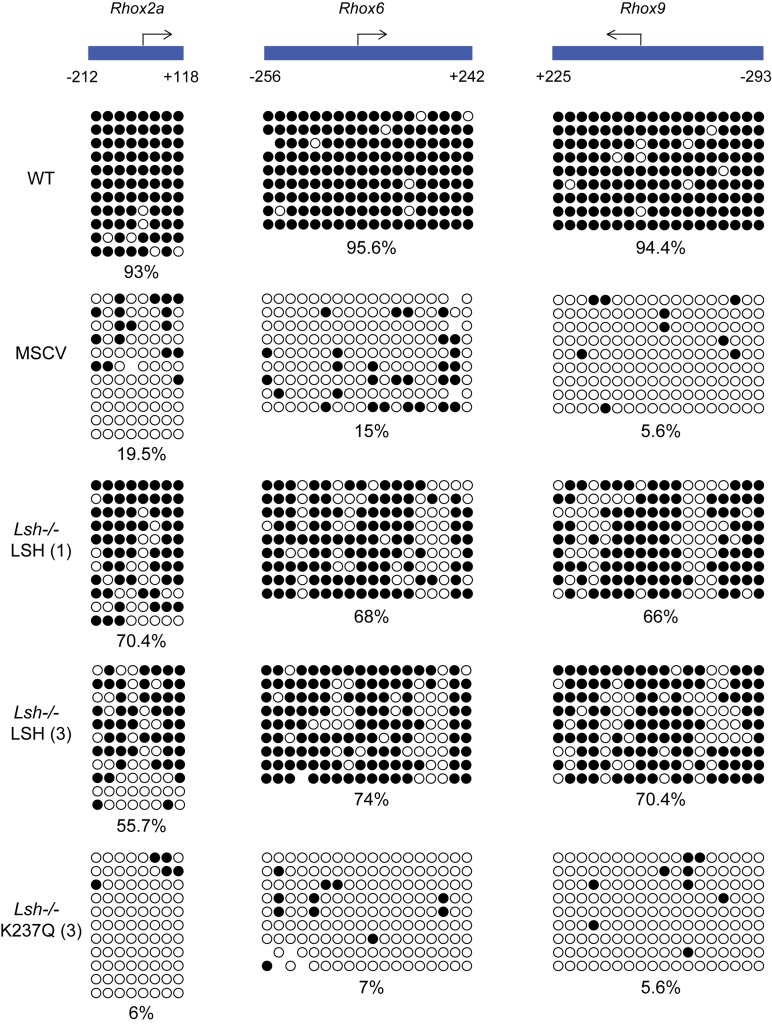
*De novo* DNA methylation of developmentally-regulated gene promoters in *Lsh−/−* MEFs expressing wild-type LSH. Bisulfite DNA sequencing of reproductive homeobox (*Rhox*) gene promoters in wild-type MEFs, *Lsh−/−* MEFs carrying an empty pMSCV vector and *Lsh−/−* MEFs expressing either wild-type or mutant LSH. The numbers below the promoter graphs indicate bp upstream (−) or downstream (+) from the transcription start site. Each row of circles is a single DNA strand and methylated CpGs are shown in black.

**Figure 4.**
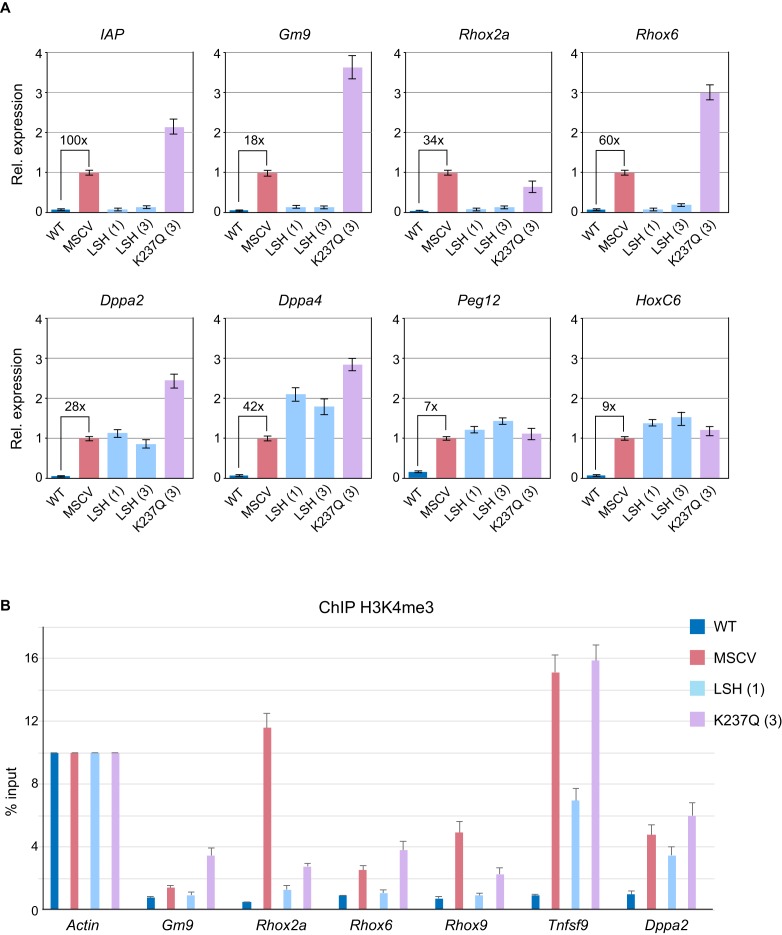
The *Lsh−/−* MEFs expressing wild-type LSH efficiently silence IAP retrotransposons and developmentally regulated genes. (**A**) Expression of IAP retrotransposons and specific genes in wild-type MEFs, Lsh−/− MEFs carrying an empty pMSCV vector and *Lsh−/−* MEFs expressing either wild-type or mutant LSH as assessed by quantitative RT-PCR. The bar graphs show fold change in expression relative to *Lsh−/−* MSCV MEFs. The housekeeping *Gapdh* gene was used for normalisation. The fold upregulation of gene expression detected by qRT-PCR in *Lsh−/−* MSCV relative to wild-type MEFs is shown numerically above the brackets. The error bars represent standard deviation. (**B**) Silencing of gene expression by wild-type LSH is accompanied by loss of H3K4me3 from gene promoters. *Tnfsf9* was randomly chosen from the list of promoters that aquire DNA methylation in the *Lsh−/−* LSH cells. The error bars represent standard deviation. A rabbit IgG was used as additional control for the anti-H3K4me3 antibody (not shown).

Given that the 5mC at the *Rhox* loci, including promoters, is established early in development following the specification of embryonic and extraembryonic lineages (10), we asked whether or not the *Lsh−/−* MEFs could methylate and silence these genes in the absence of exogenous signals. Surprisingly, bisulfite DNA sequencing detected an almost complete methylation of *Rhox2a, Rhox6* and *Rhox9* promoters in *Lsh−/−* LSH cell lines, while DNA methylation was absent in *Lsh−/−* K237Q and *Lsh−/−* MSCV cells (Figure 3). *De novo* DNA methylation of gene promoters in the *Lsh−/−* LSH cell lines was also accompanied by robust silencing of *Rhox* as well as other LSH-dependent loci, such as *Gm9* and IAP retrotransposons (Figure 4A). Transcriptional silencing and *de novo* DNA methylation of gene promoters in the *Lsh−/−* LSH cells were also accompanied by loss of histone H3 lysine 4 trimethylation (H3K4me3) (Figure 4B), a histone modification that marks active promoters and CpG islands in mammalian cells (31,32). None of the examined genes were silenced and often showed a mild increase in expression and H3K4me3 in the *Lsh−/−* K237Q MEFs (Figure 4A and B). In contrast to the *Rhox* loci, we did not observe silencing of *HoxC6, Peg12* and pluripotency genes *Dppa2* and *Dppa4* (Figure 4A). The promoters of *Dppa2* and *Dppa4* remained unmethylated in *Lsh−/−* LSH cells (Supplementary Figure S2A and B) suggesting that additional signals might be required for silencing and *de novo* DNA methylation of these loci. However, treatment of *Lsh−/−* LSH and *Lsh−/−* K237Q MEFs with retinoic acid induced silencing of *Dppa2* and *Dppa4* and methylation of promoter sequences, but this repression could not be stably maintained in *Lsh−/−* K237Q MEFs (Supplementary Figure S2C and D).

Taken together, our analyses demonstrate that, in addition to repetitive sequences, the *Lsh−/−* LSH MEFs are capable of efficient silencing and *de novo* methylation of active, H3K4me3-marked developmentally regulated promoters and that these events require ATP hydrolysis by LSH and can take place in the absence of exogenous signals.

### The catalytic activity of LSH supports *de novo* DNA methylation by both DNMT3 enzymes

**Figure 5.**
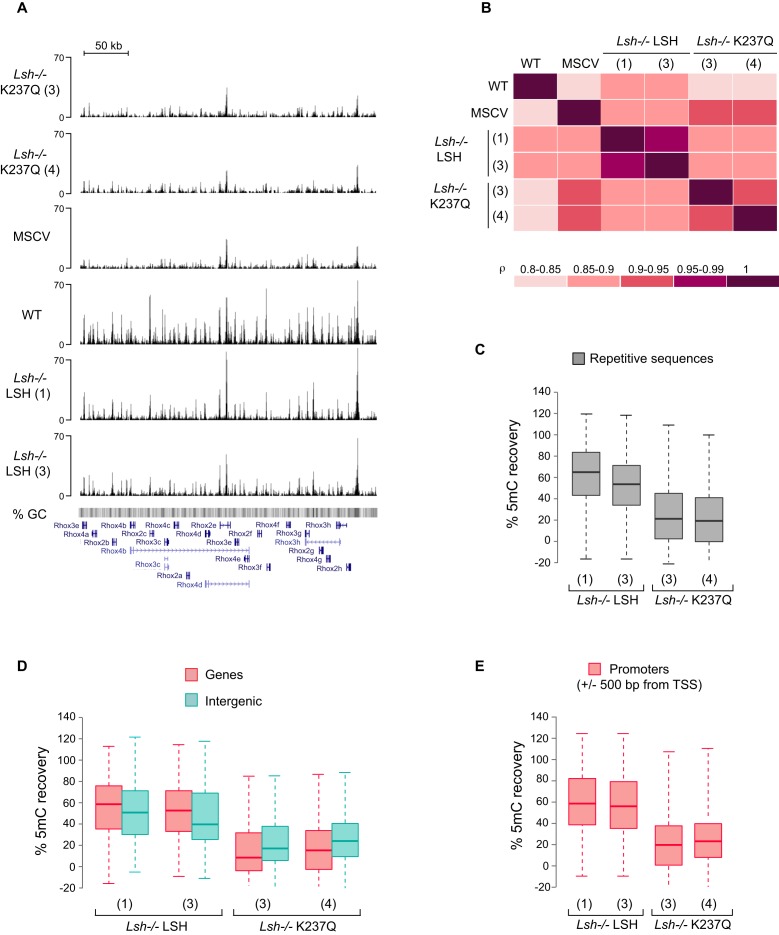
Global patterns of DNA methylation in the *Lsh−/−* MEFs expressing either wild-type or mutant LSH. (**A**) Profile of unique MeDIP-seq reads across a 330 kb region within the *Rhox* gene cluster on the X chromosome. (**B**) Spearman correlation coefficient matrix displays pairwise comparison of DNA methylation profiles between the analyzed cell lines. Boxplots display DNA methylation recovery (**C**) at repetitive sequences; (**D**) within genes and intergenic regions; (**E**) at gene promoters in the indicated cell lines. Only promoters methylated in wild-type MEFs, but not in the *Lsh−/−*MEFs were used in the graph shown in **E**. Each box in **C**, **D** and **E** represents the interquartile range (IQR) and the lowest and highest values within 1.5x IQR are shown as whiskers.

As the expression of wild-type LSH in the *Lsh−/−* MEFs restored the 5mC levels from 52% to ∼ 75% of those measured in wild-type MEFs (Figure 2A), we next asked whether or not the *Lsh−/−* LSH MEF cell lines displayed heterogeneity of DNA methylation patterns and if DNA methylation was restored uniformly across the genome. To address these questions, we carried out methylated DNA immunoprecipitation (24) followed by high-throughput sequencing (MeDIP-seq) on DNA purified from wild-type MEFs, *Lsh−/−* MSCV, *Lsh−/−* LSH (lines 1 and 3) and *Lsh−/−* K237Q (lines 3 and 4). The obtained reads were aligned to the genome and analyzed further. A visual inspection of DNA methylation patterns at specific loci, for example the *Rhox* cluster of homeobox genes (Figure 5A), and pairwise comparisons between the samples (Figure 5B) revealed that the DNA methylation patterns of both *Lsh−/−* LSH cell lines were very similar to each other and resembled those of the wild-type MEFs. As expected, the patterns of 5mC of the *Lsh−/−* K237Q cell lines showed the highest correlation with each other and the *Lsh−/−* MSCV cells. This suggests that the expression of LSH in the *Lsh−/−* MEFs results in a relatively uniform, although incomplete (Figures 2C and 3), *de novo* methylation of LSH-dependent loci.

In ES cells, DNMT3B is recruited to chromatin via binding to H3K36me3, a modification established co-transcriptionally in gene bodies, while DNMT3A displays a more broad distribution across the genome, which positively correlates with CpG density (15). In addition, both DNMT3A and DNMT3B, are capable of interacting with the unmodified histone H3 tails via a conserved ADD domain (33–35). Similar to ES cells, the calculated mean methylation of gene bodies in wild-type MEFs was higher than the median DNA methylation in the intergenic regions (Supplementary Figure S2A, WT) and methylation of both types of sequences was reduced proportionally in the *Lsh−/−* MEFs (Supplementary Figure S3A, MSCV).

To assess further if LSH supports *de novo* methylation by both DNMT3 enzymes, we calculated the mean DNA methylation recovery in gene bodies and intergenic regions for the *Lsh−/−* LSH and *Lsh−/−* K237Q cells (see Methods). The calculated recovery of DNA methylation was 40–80% for both types of regions with higher median value for gene bodies than intergenic regions (Figure 5D). Interestingly, we also detected a weak, but consistent recovery of DNA methylation in the *Lsh−/−* K237Q cell lines (Figure 5D and Supplementary Figure S3). In contrast to wild-type LSH, the median recovery values for intergenic regions were higher than those for gene bodies in LSH K237Q expressing cells (Figure 5D). This indicates that although both DNMT3 enzymes require the catalytic activity of LSH for efficient *de novo* DNA methylation, the chromatin remodeling by LSH plays a more significant role in DNMT3B- rather than in DNMT3A-dependent methylation.

Consistent with the bisulfite sequencing data for IAPs and *Rhox* promoters (Figures 2 and 3), DNA methylation was also restored with 3–4 fold higher efficiency at all classes of repetitive sequences and gene promoters in the *Lsh−/−* LSH cell lines in comparison with *Lsh−/−* K237Q cells (Figure 5C, E and Supplementary Figure S3B). Overall, 96% of all promoters that were hypomethylated in the *Lsh−/−* MEFs substantially recovered 5mC in the *Lsh−/−* LSH cell lines (Supplementary Figure S3B and C). Taken together, these data support our global and locus-specific analyses of DNA methylation (Figures 1 and 2) and demonstrate that upon LSH expression in the *Lsh−/−* MEFs *de novo* DNMTs are able to access genic, intergenic and promoter sequences.

### DNMT3B is necessary for *de novo* DNA methylation and silencing of LSH-dependent loci

**Figure 6.**
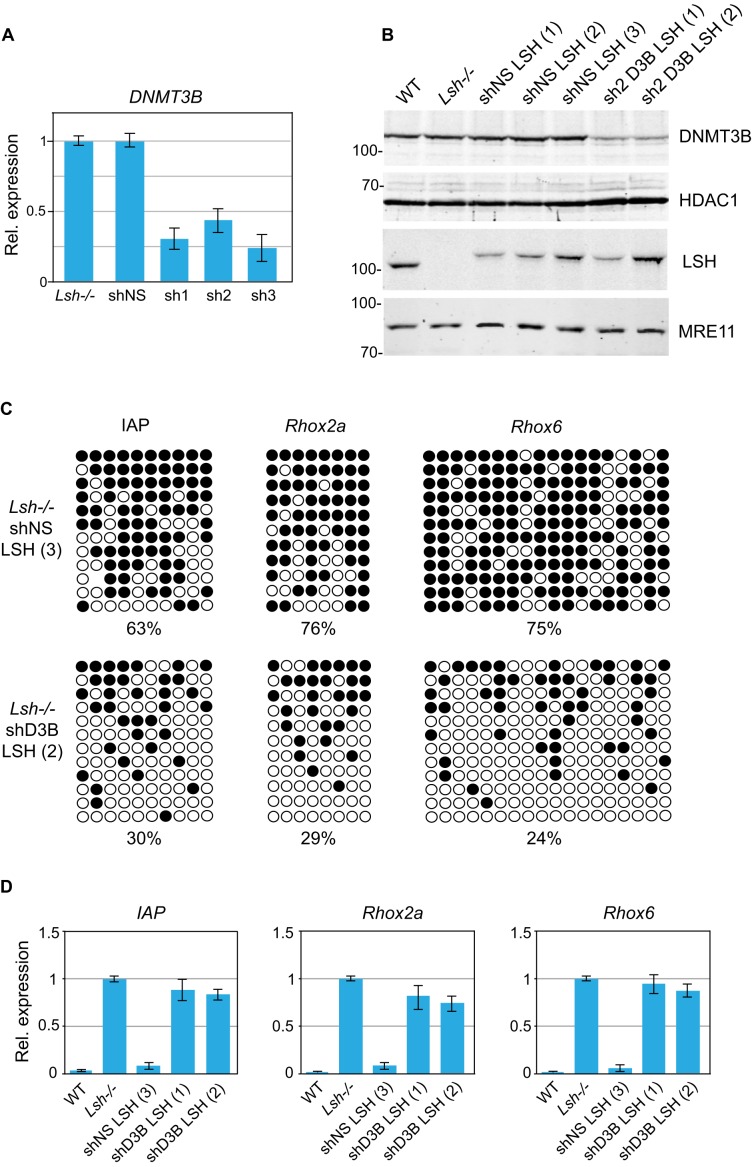
DNMT3B is required for *de novo* DNA methylation of IAPs and gene promoters upon LSH expression in *Lsh−/−* MEFs. (**A**) Expression of DNMT3B in *Lsh−/−* MEFs expressing either a non-silencing shRNA (shNS) or shRNAs (sh1, sh2 and sh3) targeting DNMT3B as assessed by quantitative RT-PCR. The error bars denote standard deviation. (**B**) Western blots showing the presence of 3xFLAG-tagged wild-type LSH in *Lsh−/−* clonal MEF lines expressing either non-silencing shRNA (shNS) or shRNA targeting DNMT3B (sh2 D3B). HDAC1 and MRE11 were used as loading controls. (**C**) Bisulfite DNA sequencing of IAPs and the promoters of *Rhox2a* and *Rhox6* genes in indicated cell lines. Methylated CpGs are shown in black. (**D**) Expression relative to *Gapdh* of *IAP*s, *Rhox2a* and *Rhox6* in indicated cell lines. The expression detected in *Lsh−/−* MEFs was designated as 1. The error bars denote standard deviation.

DNA methyltransferase DNMT3B is implicated in the establishment of DNA methylation in post-implantation embryos (6,9) as well as in *de novo* methylation of the *Rhox* loci (10) and centromeric repeats (8). As LSH directly interacts with DNMT3B and indirectly, via DNMT3B, with DNMT1 and histone deacetylases HDAC1 and HDAC2 (20), we asked if DNMT3B is responsible for methylation of repetitive sequences and unique loci in *Lsh−/−* LSH MEFs. To address this, we first stably knocked down DNMT3B in *Lsh−/−* MEFs by lentiviral vectors expressing small hairpin (sh) RNAs (Figure 6A, sh1-3). In parallel, we established a control cell line carrying a non-silencing shRNA (Figure 6A, shNS). Although all three shRNA targeting DNMT3B reduced the levels of DNMT3B mRNA and protein by 60–75% (Figure 6A and B), cells carrying the most efficient shRNAs (sh1 and sh3) displayed growth inhibition and were not used further. We next introduced the wild-type LSH into the *Lsh−/−* sh2-D3B and into the control shNS cells and subsequently established stable clonal sh2D3B-LSH and shNS-LSH cell lines (Figure 6B).

Analyses of IAPs as well as *Rhox2* and *Rhox6* promoters by bisulfite DNA sequencing and mRNA levels by quantitative RT-PCR reveled that while gene silencing and DNA methylation were established efficiently at these loci upon expression of LSH in the control *Lsh−/−* shNS MEFs, both *de novo* DNA methylation (Figure 6C) and transcriptional silencing (Figure 5D) were impaired in DNMT3B knockdown sh2D3B-LSH MEFs. These experiments demonstrate that the cellular concentration of DNMT3B is critical for LSH-mediated gene silencing and *de novo* DNA methylation in somatic cells.

## DISCUSSION

It is well established that early embryogenesis is orchestrated by morphogen gradients, signaling pathways and strong transcription factors, which shape the repertoire of gene expression in differentiating cells. A number of studies have linked chromatin modifications and DNA methylation to developmental signaling (36) and DNA methylation reprogramming to major developmental transitions from toti- and pluripotency to developmentally restricted cell fates (2,3). Given this knowledge, we asked in this study whether or not *de novo* DNA methylation can occur in somatic cells in the absence of exogenous signals.

The *Lsh−/−* MEFs are an excellent model to address this question as it is assumed that in the absence of LSH-dependent chromatin remodeling a proportion of developmentally programmed *de novo* DNA methylation events have failed to occur in embryos from which these cells are derived (18,19). Thus, we aimed to determine if restoring the LSH expression in *Lsh−/−* MEFs would result in patterns of DNA methylation that resemble those of their wild-type counterparts. This is an important question as recently mutations in LSH were found in a subset of patients with ICF (Immunodeficiency, Centromeric instability and Facial anomalies) syndrome (37), a human disease most commonly associated with congenital mutations in *DNMT3B* (38,39). Thus, addressing the reversibility of DNMT3B and LSH deficiency-caused phenotypes in differentiated cells is instrumental to motivate further research into improved therapies for ICF syndrome patients.

Surprisingly, we found that the expression of LSH in *Lsh−/−* MEFs grown in culture for many cell generations substantially reestablishes DNA methylation in the absence of additional signals. We also found that LSH supports *de novo* DNA methylation by both DNMT3 paralogues, DNMT3A and 3B, and that an appropriate cellular concentration of DNMT3B as well as the ability of LSH to bind and hydrolyse ATP are critical for efficient *de novo* DNA methylation at unique and repetitive sequences. The latter is in agreement with recent reports (25,40) and strongly suggests that chromatin remodeling by LSH is necessary for its function in DNA methylation and double-strand break repair.

*De novo* DNMTs display high affinity for chromatin (34,41) and complex binding patterns across the genome, which are determined by the CpG density and histone modifications (15). In contrast, LSH displays low affinity for DNA/chromatin and a highly dynamic behavior (42). Thus it is conceivable that the patterns of *de novo* DNMT occupancy remain unchanged in the *Lsh−/−* MEFs and that these patterns and the overall residence time of DNMTs on chromatin, which might be higher on stably positioned nucleosomes, could determine the sites where chromatin remodeling by LSH is needed for efficient *de novo* DNA methylation. Such model would explain the accurate, yet incomplete, reestablishment of DNA methylation patterns in the *Lsh−/−* MEFs upon LSH re-expression. In agreement with this, we demonstrate that that the appropriate concentration of DNMT3B is required for LSH-dependent gene silencing and *de novo* DNA methylation. Thus the partial knockdown of DNMT3B in the *Lsh−/−* MEFs prior to LSH expression impaired both silencing and *de novo* methylation of LSH-dependent loci. Since the expression of DNMT3A and 3B, but not DNMT1, is downregulated upon differentiation of embryonic cells (9,19), the low concentration of *de novo* enzymes as well as the absence of DNMT3L may limit the extent of LSH-mediated reestablishment of DNA methylation in somatic cells. We attempted to overexpress either DNMT3A and 3B in *Lsh−/−* LSH MEFs, but were unable to isolate stable clones, which could indicate that somatic cells do not tolerate elevated expression of *de novo* DNMTs.

Importantly, the LSH-induced *de novo* DNA methylation in *Lsh−/−* cells occurred at inactive as well as active, H3K4me3-marked, promoters resulting in stable silencing of associated genes and transposons, as demonstrated here for the *Rhox* loci and IAPs. This raises the question of how LSH and *de novo* DNMTs are recruited to active promoters. As the binding of DNMT3A and 3B to the N-terminus of H3 is inhibited by the presence H3K4 di- and trimethylation (34,41), it seems unlikely that the recruitment of LSH to active promoters is directed by either of the two DNMT3 paralogues. Addressing this question requires further studies in an inducible system that would enable controlled re-expression of LSH in the *Lsh−/−* MEFs and detailed investigation of chromatin dynamics, transcriptional silencing and the establishment of *de novo* DNA methylation following LSH induction. In parallel, a better characterization of LSH-associated proteins may also shed light on the mechanisms that govern the establishment of LSH-dependent heterochromatin.

## Supplementary Material

SUPPLEMENTARY DATA
