# Supplementary material for: The SNF2 family ATPase LSH promotes cell-autonomous de novo DNA methylation in somatic cells
Source: Nucleic Acids Res. 2016 May 13;44(16):7592–604. doi: 10.1093/nar/gkw424 (PMC5027476; doi:10.1093/nar/gkw424)
Supplement: SUPPLEMENTARY DATA [file supp_44_16_7592__index.html]

The SNF2 family ATPase LSH promotes cell-autonomous de novo DNA methylation in somatic cells — SUPPLEMENTARY DATA 

# The SNF2 family ATPase LSH promotes cell-autonomous *de novo* DNA methylation in somatic cells

## SUPPLEMENTARY DATA

- SUPPLEMENTARY DATA
