# Supplementary material for: The SNF2 family ATPase LSH promotes cell-autonomous de novo DNA methylation in somatic cells
Source: Nucleic Acids Res. 2016 May 13;44(16):7592–604. doi: 10.1093/nar/gkw424 (PMC5027476; doi:10.1093/nar/gkw424)
Supplement: SUPPLEMENTARY DATA [file supp_gkw424_nar-00448-v-2016-File008.pdf]

Supplemental Information

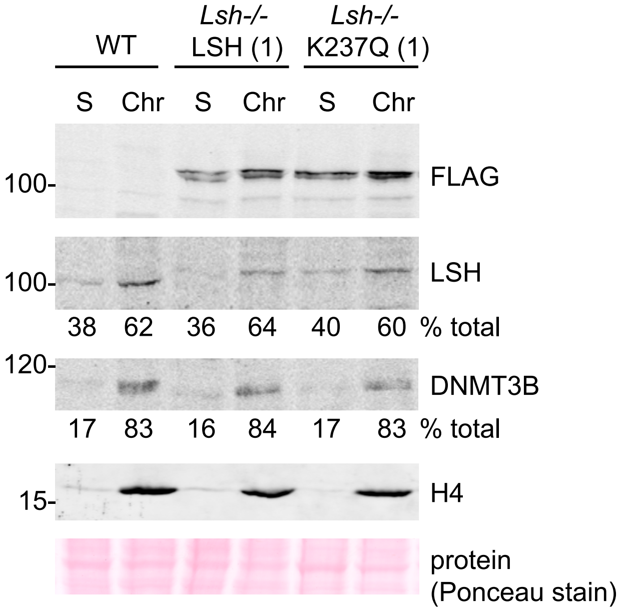

Figure S1 The ATP binding site mutation does not disrupt the association of LSH with chromatin

Soluble nuclear proteins (S) were extracted with 200 mM NaCl, 0.1% Tween followed by benzonase digestion and extraction of chromatin associated proteins (Chr) with 500 mM NaCl. The soluble and chromatin associated proteins (60 μg) were resolved in either 8.5% or 15% (to detect histones) SDS-PAGE and the Western blots were probed with the indicated antibodies and IRDye conjugated secondary antibodies. The blots were imaged on LiCOR Odyssey scanner and the signals quantified by Image Studio software. The numbers shown below each lane indicate % of total LSH and DNMT3B. Note that the K237Q mutation does not disrupt the association of LSH and DNMT3B with chromatin in MEFs.

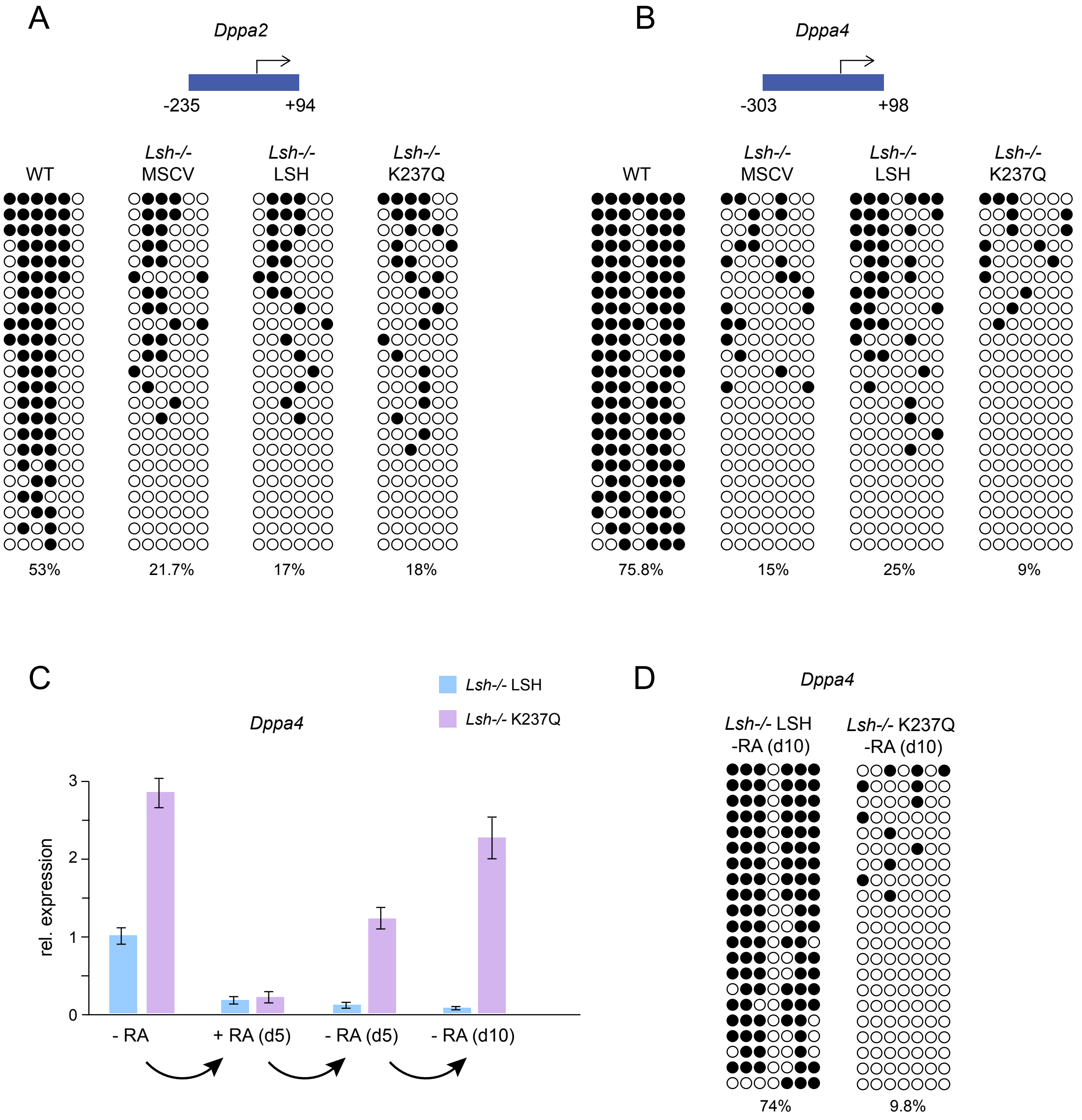

Figure S2 ***Lsh-/-*** **MEFs expressing wild-type LSH require additional signals for silencing of pluripotency-associated genes**

Bisulfite DNA sequencing of *Dppa2* (**A**) and *Dppa4* (**B**) promoters in wild-type MEFs, *Lsh-/-* MSCV MEFs and *Lsh-/-* MEFs expressing either wild-type or K237Q mutant LSH. Methylated CpG are indicated with black circles and % methylation is shown below the diagrams. The graphs above the bisulfite sequencing diagrams show the span of the sequenced regions relative to the transcription start site (arrow). (**C**) Treatment of *Lsh-/-* MEFs expressing either wild-type or mutant LSH with 300 nM retinoic acid for 5 days leads to silencing of *Dppa4*. However, this silenced state cannot be stably maintained by cells expressing the K237Q mutant LSH. The graph shows the expression of *Dppa4* relative to GAPDH as measured by quantitative RT-PCR. The error bars represent standard deviation calculated from 2 biological replicates with 3 technical replicates carried out for each time point. (**D**) Bisulfite DNA sequencing of *Dppa4* promoter 10 days after removal of retinoic acid from the culture. % methylation is shown below the diagrams.

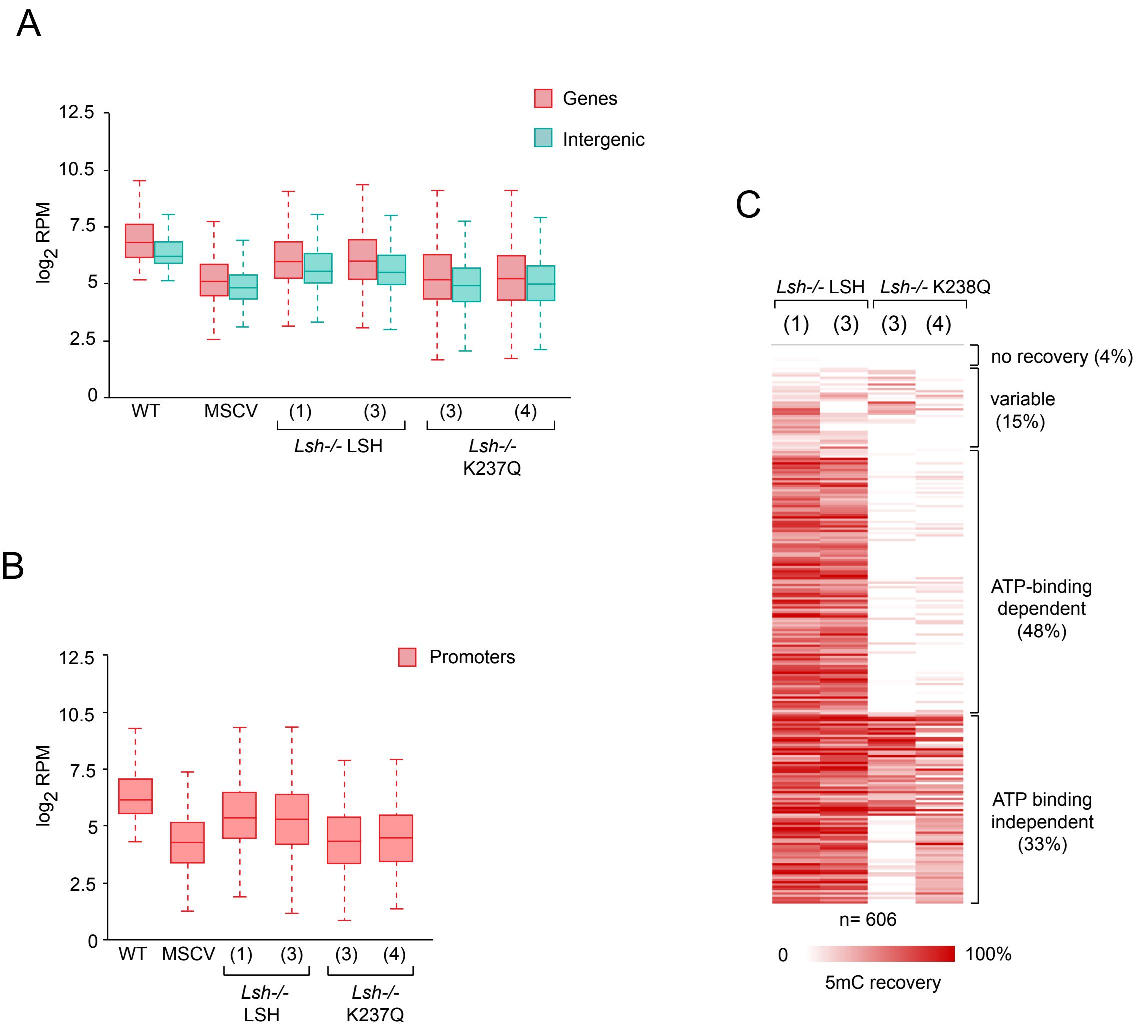

Figure S3 Global and locus specific recovery of DNA methylation in the *Lsh-/-* MEFs expressing wild-type and mutant LSH

Median number of reads for (**A**) genes and intergenic sequences and (**B**) promoters (+/- 500 bp from TSS) obtained from the normalised MeDIP-seq data for all six analysed cell lines. The boxes denote the interquartile range (IQR) and the lowest and highest values within 1.5x IQR are shown as whiskers. (**C**) A heat map showing 5mC % recovery at individual promoters. Note that 15-33% of promoters show ATP binding-independent recovery in *Lsh-/-* K238Q cell lines. This recovery, however, is below 50% and much weaker than in the *Lsh-/-* LSH cell lines. “n” represents the number of promoters. Only loci showing >3 fold difference in methylation between WT and MSCV cells were considered in these analyses.

Table 1 List of primers

A. Bisulfite DNA sequencing

Locus	Forward	Reverse
IAP	GGCGTTGATAGTTGTGTTTTAAGTGGTAAAT	ATTCTAATTCTAAAATAAAAAATCTTCCTTA
Rhox2	TTTTTAGTGAATTGTTATTTTTTTT	ATCCCACATCAAACTTATAATTAAC
	TTTTTTATTGTTTTGAGTTTTGTAG	ATCCCACATCAAACTTATAATTAAC
Rhox6/9	GTTTGAGGAGGTTTTTGTGTTATTT	AAATCCAATCAAAACCAATTTACTC
	TTATTTTTTGTGAAGGAATTTGAAT	AAATCCAATCAAAACCAATTTACTC
Dppa2	TGTTATGTTTTGTTTTGGATTTAAAA	AAAAAAACCAATTCTCTTAAAAAATCA
	TGTTATGTTTTGTTTTGGATTTAAAA	TCCTATAACCCAATTAAACTCTTCC
Dppa4	AGTTGTTAGGAGTAGGGGGTAGTAGTT	AAAACCCTCATTTAAAAAACCAAAT
	AGTTGTTAGGAGTAGGGGGTAGTAGTT	TCCAACAATCTCCATCTTAAAAATAA

B. Quantitative RT-PCR

Transcript	Forward	Reverse
GAPDH	GGCTCATGACCACAGTCCATGCC	CACGGAAGGCCATGCCAGTGAG
IAP	ACTAACTCCTGCTGACTGG	TGTGCTTGCTCATAGATTAG
Gm9	GCTGAATCCGTTGGGCACAGTGAA	GGAGGTAAAGGTCGTTGGGAGCCA
Rhox2a	CACTAAGGAGGCAAATCGCCTGGCA	ACAGCCTTCACAGAGAGATGCTCCG
Rhox6	CAGCTTGCGAGCAAGGAGGGATCT	ATCTTCAGGGAGAGTCGCTCTGGG
Dppa2	GCATTCATTCAGCGGCTGCCTTT	TGCGTAGCGTAGTCTGTGTTTGG
Dppa4	CAAGGGCTTTCCCAGAACAAATGC	GCAGGTATCTGCTCCTCTGGCAC
Peg12	TCGGGAAACCTCATCAAGGAGGCG	GTTCTGAGCTGTGCCCTCCATCAC
HoxC6	GAATGAATTCGCACAGTGGGGTCG	TTTGATCTGTCGCTCGGTCAGGCA

C. Chromatin Immunoprecipitation

Promoter	Forward	Reverse
ActinB	ATGAAGAGTTTTGGCGATGG	GATGCTGACCCTCATCCACT
Gm9	TTTCTCTATAGCAGCCCAAGTACC	AAGCGCCTAAAGTTTCAAAGACAC

Rhox2	GAATAAGGACTTCCACGGCTTTAC	TCCAGTTCAATTCTATATGGGATGC
Rhox6	CCATGTTGCTCAGGTCTTTATCTC	GAGCGAGCCAGTTCAGTACAAG
Rhox9	GAGCAAGCCCGCTCATAACAAG	GCCATGATGCTCAGATCTTTATCTC
Tnfsf9	CTCTATGGCCTAGTCGCTTTGG	GTGACCTGGTCTGCATTATTCTC
Dppa2	AAAAGAAGTCGGCATTCATTCAGC	AGCCCTAGAGTGAAAAAGAACTCC
